# Copy Number Variation in *SOX6* Contributes to Chicken Muscle Development

**DOI:** 10.3390/genes9010042

**Published:** 2018-01-17

**Authors:** Shudai Lin, Xiran Lin, Zihao Zhang, Mingya Jiang, Yousheng Rao, Qinghua Nie, Xiquan Zhang

**Affiliations:** 1 Guangdong Provincial Key Laboratory of Agro-Animal Genomics and Molecular Breeding, Key Laboratory of Chicken Genetics, Breeding and Reproduction, Ministry of Agriculture, College of Animal Science of South China Agricultural University, Guangzhou 510642, China; shudailin@stu.scau.edu.cn (S.L.); daanxiran@163.com (X.L.); jayhozhang@163.com (Z.Z.); jmyykl@stu.scau.edu.cn (M.J.); nqinghua@scau.edu.cn (Q.N.); 2 Department of Biological Technology, Nanchang Normal University, Nanchang 330029, China; rys8323571@aliyun.com

**Keywords:** chicken, copy number variation, CNV, SOX6, growth, skeletal muscle, disorder region

## Abstract

Copy number variations (CNVs), which cover many functional genes, are associated with complex diseases, phenotypic diversity and traits that are economically important to raising chickens. The sex-determining region Y-box 6 (*Sox6*) plays a key role in fast-twitch muscle fiber differentiation of zebrafish and mice, but it is still unknown whether *SOX6* plays a role in chicken skeletal muscle development. We identified two copy number polymorphisms (CNPs) which were significantly related to different traits on the genome level in chickens by AccuCopy^®^ and CNVplex^®^ analyses. Notably, five white recessive rock (CN = 1, CN = 3) variant individuals and two Xinghua (CN = 3) variant individuals contain a CNP13 (chromosome5: 10,500,294–10,675,531) which overlaps with *SOX6*. There is a disordered region in SOX6 proteins 265–579 aa coded by a partial CNV overlapping region. A quantitative real-time polymerase chain reaction showed that the expression level of SOX6 mRNA was positively associated with CNV and highly expressed during the skeletal muscle cell differentiation in chickens. After the knockdown of the SOX6, the expression levels of IGFIR1, MYF6, SOX9, SHOX and CCND1 were significantly down-regulated. All of them directly linked to muscle development. These results suggest that the number of CNVs in the CNP13 is positively associated with the expression level of SOX6, which promotes the proliferation and differentiation of skeletal muscle cells by up-regulating the expression levels of the muscle-growth-related genes in chickens as in other animal species.

## 1. Introduction

Copy number variations (CNVs) are defined as large DNA fragments with sizes ranging from 50 base pairs (bp) to 3 megabases (Mb) deleted, inserted, duplicated, or translocated in the genome [1], and are a kind of important genomic structure variation. The same region adjacent to and partially overlapping multiple CNVs can be incorporated into a large genomic segment called the copy number variation region (CNVR). A CNVR with a frequency of at least 3% is defined as copy number polymorphism (CNP) [2]. The current research into complex diseases or phenotypic traits is mainly concentrated on association studies with single nucleotide polymorphisms (SNPs), relatively few association studies focus on CNV [3]. With the construction of the chicken genome CNV sketches of different breeds, the researchers also found many phenotypic traits associated with chicken CNVs. For example, the chicken’s slow feathering is relative to the partial duplication between the *PRLR* and *SPEF2* genes in the K loci of the chromosome (chr) Z [4]. The chicken’s bean crown is caused by a large amplification fragment near the first intron of the sex-determining region Y-box 5 (*SOX5*) [5]. Visceral pigment deposition in Silkies is caused by the repetitive regions in the endothelin 3 (*EDN3*) gene [6]. A few CNVs are also associated with chickens’ resistance or susceptibility to Marek’s disease [7]. Therefore, the identification of CNV plays an important role in the research into some phenotypic traits and the process of disease susceptibility, and it is an important reference value for early breeding in chickens. 

It has been reported that *SOX6*, one of the sex-determining region Y family members, plays essential roles in sex determination, embryonic and nervous system development and the formation of skeletal and various organs [8]. The transcription factors of the Sox family interact with each other and play a pivotal role in the target organ. For example, SOX5 and SOX6 factors directly or indirectly act on SOX9, and then regulate the proliferation and differentiation of chondrocytes [9,10], cartilage formation and skeletal development [11]. Sox6 is a multi-faceted transcription factor involved in the terminal differentiation of many different cell types in vertebrates. It has been suggested that in mice as well as in zebrafish, Sox6 plays a pivotal role in the terminal differentiation of skeletal muscle as a key regulator of fast-twitch muscle fiber differentiation [12] or affecting the expression of sarcomere protein genes, as well as an indirect role through influencing the expression of transcription factors relevant to muscle development [13,14].

Recently, according to the analysis of chicken CNV data [15], we found that the chicken *SOX6* overlaps in the CNP13. We propose that this *SOX6’s* CNVR will directly or indirectly affect chicken growth and development through changing the expression level of SOX6. Thereby, in this study, we aim to study how the CNV influences the expression level of SOX6, and how SOX6 regulates chicken skeletal muscle development. Hence, we used association analysis between the CNP and economically important traits for raising chickens, and the targeted overexpression and interference of the SOX6.

## 2. Materials and Methods

### 2.1. Ethical Approval

The Animal Care Committee of South China Agricultural University (Guangzhou, China) approved this study (project code 2015-A003, approval on 18 May 2015). The animals involved in this study were humanely sacrificed as necessary to ameliorate suffering.

### 2.2. Experimental Animals

White recessive rock (WRR) is a fast-growing broiler line that was bred for meat, while Xinghua (XH) is a Chinese native breed with slower growth, lower production, and more favorable meat quality. Three each of XH and WRR chickens at 7 weeks of age and three each of XH and WRR chickens at 27 weeks of age (12 total) were humanely euthanized and necropsied to collect tissue samples. First, scissors were used to cut open a cerebral longitudinal fissure and a cerebellar horizontal fissure, remove the brain meninges, and then the following samples were collected: brain (cerebral hemisphere), cerebellum, hypothalamus (in ventral thalamus) and pituitary (at the bottom of a brain; its anterior lobe and posterior interlobar separated by a connective tissue sheath). Secondly, the skin was cut carefully from the middle of the chest to the abdomen, and then the following samples were collected: sebum (subcutaneous chest fat), breast muscle (pectoralis major) and leg muscle (gastrocnemius). Thirdly, the viscus was explored using scissors to cut open the ribs and sternum, and then the following samples were collected: abdominal fat (fat around the gizzard), heart (pericardium, fat and arteriovenous removal), liver (right hepatic lobe), spleen (capsule removal), lung (spongy architecture and closed to the chest wall), kidney (garnet-red and located on both sides of the lumbosacral cavity), glandular stomach (end of esophagus, with many papillary protuberances on the surface of the inner wall), gizzard (elliptical and double convex mirror shape, content and corneum membrane of the inner wall removal), and ovary (connected to the oviduct, elliptical and mulberry). About 0.5 g of each tissue sample was collected and put in 2 mL frozen pipes, and quickly thrown into liquid nitrogen containers. All samples were stored at −80 °C in a refrigerator for a long time. They were used to analyze the tissue expression patterns of candidate genes. The XH and WRR chickens were provided by the South China Agricultural University Poultry Breeding Farm (Guangzhou, China) and Guangdong Wen’s Nanfang Poultry Breeding Co. Ltd. (Yunfu, China), respectively.

The genomic DNA was prepared from venous blood samples using a blood DNA extraction kit (Omega, Irving, TX, USA). Total RNA was extracted using Trizol reagent (Invitrogen, Carlsbad, CA, USA).

### 2.3. CNV Population Genetics

The SNPs were genotyped using a chicken 60 K SNP Illumina iSelect chicken array (Illumina, Towne Centre Drive San Diego, CA, USA) and collected according to previous published studies by Rao et al. [15] and Xie et al. [16] from 554 XH and WRR full-sib family chickens (33 F0 + 32 F1 + 489 F2 individuals) and were used to analyze each groups’ CNP and the correlation between a candidate CNP and the economic traits.

The information on each CNVR for a family that contains family and individual CNV information can be represented in a specific genotype, such as 0/0 (CN = 0, homozygous deletion), 0/1 (CN = 1, single copy deletion), 1/1 (CN = 2, normal copy), 0/2 (CN = 2, multi-bit complex CNV), 1/2 (CN = 3, single copy increase) and 0/3 (CN = 3, multi-bit complex CNV). The CNV inheritance in a family can be analyzed according to the CNV types of F0, F1 and F2 generations in the same CNVR of the same chromosome.

### 2.4. Association Analysis

The frequency of each CNVR group was calculated for each growth, carcass, and quality trait as follows: the growth traits recorded in the F2 generation included the weight at birth and at 7, 14, 21, 28, 35, 42, 49, 56, 63, 77, 84 days of age; shank length at 42, 49, 56, 63, 77, 84 days of age; tibial diameter at 42, 49, 56, 63, 77, 84 days of age; and daily gain from 0–4 weeks of age and 4–8 weeks of age. The carcass traits are carcass weight, half eviscerated weight, eviscerated weight, brisket weight, leg weight, wing weight, head and neck weight, tibia claw weight, abdominal fat weight, abdominal fat belt width, subcutaneous fat thickness and fat septa width. The meat quality traits evaluated are muscle color, pH value, electrical conductivity, shear force, muscle fiber cross-sectional area, the department of hydraulic, intramuscular fat content, crude protein content. The generalized linear model (GLM) program of the SAS9.3 software (SAS Institute Inc., Cary, NC, USA) was used to analyze the association between each CNP and these three kinds of traits. The linear model used was as follows:

Yijkl = µ + Gi + Sj + Hk + Fl + eijkl,(1)

where *Yijkl* = corrected phenotypic value, *μ* = common mean, *Gi* = CNV genotypic effect, *Sj* = fixed gender effect, *Hk* = fixed hatch effect, *Fl* = random family effect, and *eijkl* = random residual. The false discovery rate (FDR) method was used for verifying significant CNPs. The *p* values of a single CNP with each trait were sorted in ascending order, and the following formula was applied to obtain the FDR for each CNP = mP(i)/i, in which *m* is the total number of traits, and *P* is the *p* value of the *i*th trait [17].

### 2.5. The Transformation of the Copy Number Polymorphism Regional Database

The CNVRs from the WuGSC2.1 database version (May 2006 (WUGSC 2.1/galGal3)) [15] were positioned to the chicken genome of the Gallus_gallus-4.0 version using LiftOver tool software (http://genome.ucsc.edu/cgi-bin/hgLiftOver), comparatively analyzing with the National Center for Biotechnology Information (NCBI, https://www.ncbi.nlm.nih.gov/) Basic Local Alignment Search Tool (BLAST, http://blast.ncbi.nlm.nih.gov/Blast.cgi) to obtain the corresponding physical location of the CNP in the new database.

### 2.6. Homology Analysis of Genes’ Coding Sequences and Construction of Phylogenetic Tree

The cDNA sequences of the SOX6 of the 12 species (*Gallus gallus* NP_001305380.1, *Homo sapiens* NP_001139291.1, *Pan troglodytes* XP_003312980.1, *Canis lupus familiaris* XP_005633754.1, *Bos Taurus* NP_001178347.1, *Mus musculus* NP_001264255.1, *Rattus norvegicus* NP_001019922.1, *Xenopus tropicalis* NP_001116887.1, *Danio rerio* NP_001116481, *Ovis aries musimon* XP_011992280.1, *Coturnix japonica* XP_015719574.1, *Equus caballus* XP_014597307.1) were obtained from the NCBI GeneBank. These sequences were download in FASTA format files and saved as txt format.

BLAST was used to analyze the homology of cDNA sequences among different species. SOX6 protein sequences of different species were mapped by using MEGA6.0 software [18] to construct a phylogenetic tree.

### 2.7. Bioinformatics of SOX6

Homologous construction of the tertiary structure of the SOX6 proteins was carried out by Swiss Model [19]. The SOX6 amino acid sequence was analyzed by GlobPlot online software [20] to predict the disordered region.

### 2.8. Detection of DNA Copy Number Variations

Both AccuCopy^®^ (based on multiplex competitive amplification) [21] and CNVplex^®^ (based on the multiplex ligation-dependent probe amplification) [22] assays developed by Genesky BioTech (Shanghai, China) were used to genotype six and four CNPs of 200 samples (100 XH chicken and 100 WRR chicken, male and female in half), respectively. The absolute copy number rounding principle was one (0.8–1.2), two (1.6–2.4), and three (2.6–3.4).

The copy number was calculated using the 2 ^−∆∆Ct^ relative quantitative method, and the formula was as follows:

ΔΔCt = ΔCt _(test)_ − ΔCt _(reference)_, NR = 2 ^−∆∆Ct^ (2)

where *test* is the samples to be tested (samples that had a CNV within the region predicted by the microarray data); *reference* is the negative sample (with no copy number variation in the analyzing region); the *NR* value of normal individuals was about one, of copy number gain individuals ≥1.5, and of one copy loss individuals ≤0.5 [23].

### 2.9. Cell Culture

In the study of muscle development, the skeletal muscle cell line, quail muscle clone 7 (QM7), is the ideal host for studying the expression of foreign genes, since it is a serum-inducible myogenic cell line. The cells replicate as myoblasts in medium containing serum. When switched to medium low concentration or without serum, the cells cease dividing and fuse to form large multinucleated myotubes. QM7 cells are useful for studying many aspects of muscle differentiation and gene expression. They also have the characteristics of easy cultivation, proliferation and differentiation, and a high transient transfection efficiency. Furthermore, the homology of the *SOX6* gene in quails and chickens was 99% (Supplementary File 1, Table S1).

Quail muscle clone 7 were cultured in 79.5% M199 culture medium (Gibco, Grand Island, NY, USA) with 10% fetal bovine serum (Hyclone, Logan, UT, USA), 10% tryptose phosphate broth solution (Sigma-Aldrich, St. Louis, MO, USA) and 0.5% penicillin/streptomycin (Invitrogen). Compared with the above, not everything changes, but 2% fetal bovine serum (Hyclone, Logan, UT, USA) induces QM7 cells to differentiate. Cells were cultured at 37 °C in an incubator with 5% CO_2_.

### 2.10. RNA Interference

QM7 cells were transfected with 50 nM of small interfering RNAs (siRNAs) targeting SOX6 (536–542 aa) using a Lipofectamine^®^ 3000 Transfection Kit (Invitrogen) according to the manufacturer’s protocol. Total RNA was harvested 48 h later for quantitative real-time PCR (qRT-PCR) analysis. The siRNA sequences of negative control (si-NC) were: sense: 5′-UUCUCCGAACGUGUCACGUTT-3′, antisense: 5′-ACGUGACACGUUCGGAGAATT-3′; and sense: 5′-GCAGAAGGAAGUAAAGCAATT-3′, antisense: 5′-UUGCUUUACUUCCUUCUGCTT-3′ for siRNA-SOX6 (GenePharma Co.; Ltd., Shanghai, China).

### 2.11. Reverse Transcription and Quantitative Real-Time PCR

The reverse transcription reaction was performed to analyze the expression levels of genes in the CNP area using the PrimeScript RT reagent kit (perfect real time) (Takara, Otsu, Japan) as described in the manufacturer’s protocol. The qRT-PCR was conducted in the Bio-rad CFX96 Real-Time Detection system (Bio-rad, Hercules, CA, USA) using a KAPA SYBR FAST qPCR Kit (KAPA Biosystems, Wobrun, MA, USA). All primers are listed in Supplementary File 1, Table S2. The expression levels of the genes both in the CNP area or not were normalized with *PCCA* or *β-actin*.

### 2.12. Cell Cycle Distribution Analysis

In order to determine the effects of SOX6 on the cell cycle distribution, QM7 cells were cultured in 12-hole plate. When cell density reached 70%, the overexpression vector (pcDNA3.1-SOX6), empty vector (pcDNA3.1), siRNA (si-SOX6) and si-NC were transiently transfected into the cells, with 4 repetitions in each group. All cells were sorted using flow cytometry at 48 h after transfection. Briefly, the cells were washed twice with ice-cold phosphate buffered saline (PBS) and fixed with 70% ice-cold ethanol overnight at −20 °C until further processing. After incubation in 50 μg/mL propidium iodide (Sigma-Aldrich) containing 10 μg/mL RNase A (Takara, Otsu, Japan) and 0.2% (*v*/*v*) Triton X-100 (Sigma-Aldrich) at 4 °C for 30 min, the cells were analyzed using a FACSAria II flow cytometer (BD Biosciences, San Jose, CA, USA) and ModFit Lt 4.1 software (Verity Software House, Topsham, ME, USA).

### 2.13. Statistical Analysis

Data were processed using the statistical software package SAS 9.1.3 (SAS Institute Inc.) and expressed as the mean ± standard deviation (SD). A variance analysis was completed using a GLM procedure. *P* < 0.05 was considered as a significant difference between the groups by *t*-test and one-way analysis of variance (ANOVA).

## 3. Results

### 3.1. Population Genetics of Chicken Copy Number Variations

From the analysis of nine chicken families, we found that there were 34 CNVRs belong to CNPs. Twenty CNVRs were consistent with Mendel’s law of segregation in these three generations, and the remaining area CNVs in some individuals were not. Six CNVRs occurred in two chicken families (Supplementary File 1, Tables S3–S11. Fortunately, we uncovered 19 CNPs with a CNP frequency from 3.1% to 10%, and 10 of them contained genes (Table 1).

Combining the genotype frequency of the candidate CNPs, which was detected by three kinds of method (Table 2), with the genetic regularity analysis, we found that some CNPs were unique in the family. For example, CNP2 and CNP4 were found only in the XH × WRR full sib resource F2 group. Repetitive events (CN = 3) are basically inherited and can be inherited in a stable manner to the next generation, such as CNP10.

### 3.2. Correlation between CNP and Chicken Economically Important Traits

As shown in Table 3, three traits were strikingly associated with CNPs. The contribution rates of these CNPs to growth traits accounted for a range from 25% to 52%. Among the 19 CNPs, CNP14 was significantly correlated with breast muscle conductivity and breast muscle dry matter content. Furthermore, CNP17 (deletion) showed a promotion effect on growth. However, there were no genes in these two regions (Table 1). Though CNP13 was not remarkably related to leg muscle fiber cross-sectional power (*p* = 0.0595), breast muscle fiber cross-sectional power (*p* = 0.074), leg muscle dry matter content (*p* = 0.085) or other economically important traits for raising chickens, it overlaps with the chicken *SOX6* gene (Table 1). It was reported that *Sox6* plays an important role in the fast-twitch muscle fiber differentiation of zebrafish and mice [12,13,14]. We want to know whether this gene acts on chicken muscle development or not.

### 3.3. Candidate Genes in CNPs Related to Chicken Growth Traits

Among the 12 CNPs associated with growth traits, the CNVRs of CNP2, CNP3, CNP4, CNP6 and CNP13 contain functional genes. Such CNPs will be considered a candidate CNP (Table 4). Thereby, there were six CNPs considered as functional candidate CNPs.

**Figure 1:**
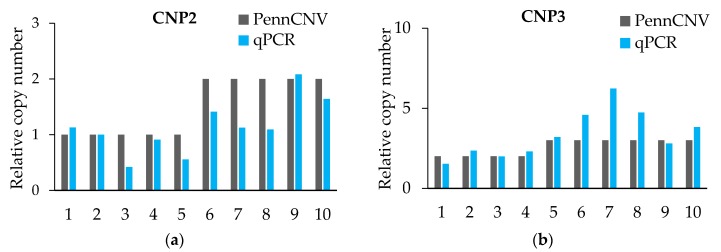

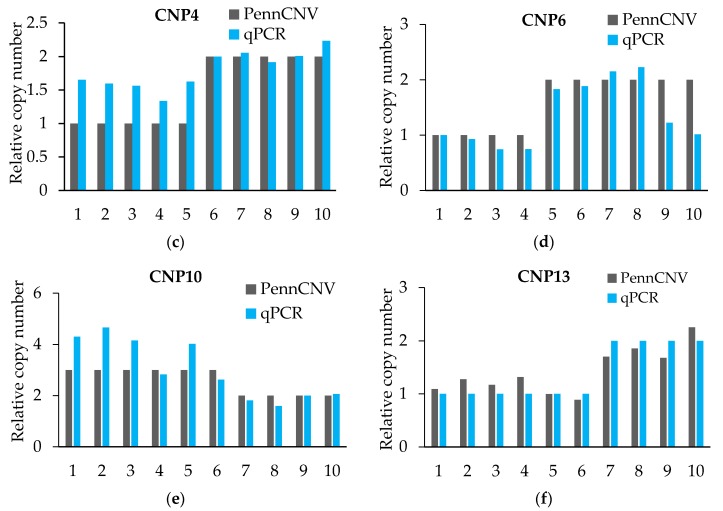
Quantitative real-time PCR (qRT-PCR) verified six CNPs. (**a**–**f**) The expression levels of CNPs are related to that of the control gene *PCCA*. PennCNV is a hidden Markov model (HMM)-based approach, for the kilobase-resolution detection of CNVs from Illumina high-density single nucleotide polymorphisms (SNP) genotyping data [15].

The qPCR validation results (Figure 1 and Table 5) initially proved the true existence of CNVs, and for more than 80% of the individuals’ CNV type was proven. However, the qPCR results were not exact in the quantification of the absolute copy number of each individual. The CNV of each CNP formed haplotype blocks with neighboring SNPs. However, we did not find a strong linkage between CNV and SNP from the linkage disequilibrium (LD) analysis, which means that we did not find out the tag-SNP of the CNPs (Supplementary Figure S2).

### 3.4. The CNV in CNPs

**Figure 2:**
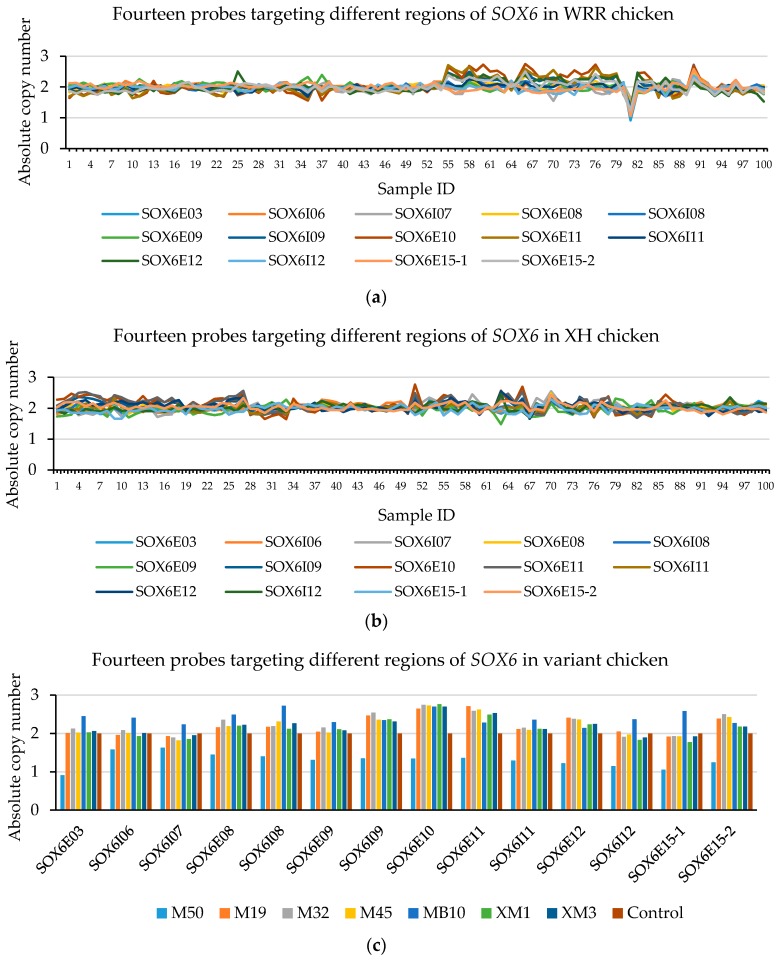
The CNVplex^®^ assay result for *SOX6* (CNP13). The different colors represent the individual’ absolute copy number of *SOX6* in the different positions in the CNP13. There are two XH individuals with absolute copy values in exons 10 and 11 of *SOX*6 of about 2.6 (CN = 3) (sample ID 51 and 66 shown in (**a**), which consist with XM1, XM3 in (**c**)); others were normal individuals (CN = 2). In WRR chickens, there is a single individual with absolute copy number less than 1.2 (CN = 1) (sample ID 81 shown in (**b**), which consists with M50 in (**c**)), and four individuals with an absolute copy values in exons 10 and 11 of *SOX6* greater than 2.5 (CN = 3) (sample ID 55, 66, 76 and 90 shown in (**b**), which consist with M19, M32, M45 and MB10 in (**c**), respectively).

From the AccuCopy^®^ assay result (Table 6), both CNP3 and CNP10 have a high sequence homology that is unable to gain the rounding number. There are no CNVs in glucocorticoid-induced transcript 1 (*GLCCI1*) (CNP2), cAMP-regulated phosphoprotein (*ARPP19*) (CNP4), and RAB40B, members of the RAS oncogene family (*RAB40B*), a family with sequence similarity among 214 member A (*FAM214A*) (CNP6). However, heterozygous deletion in *SOX6* (CNP13) was found in WRR chickens. From the CNVplex^®^ assay results, among 100 WRR and 100 XH chickens, there were no individuals with CNVs in the myosin VA (*MYO5A*) (CNP4) and WD repeat domain 45B (*WDR45B*) (CNP6), while there were two XH and two WRR chickens with CN = 3 of *SOX6* (CNP13) (Figure 2, Supplementary File 2), five WRR hens with CN = 1 or CN = 3, and five XH individuals with CN = 3 in CNP10 (Supplementary File 1, Figure S3). Thus, we focused on *SOX6* (CNP13) in the next study.

### 3.5. The CNVs in CNP Structural Mapping

**Figure 3:**
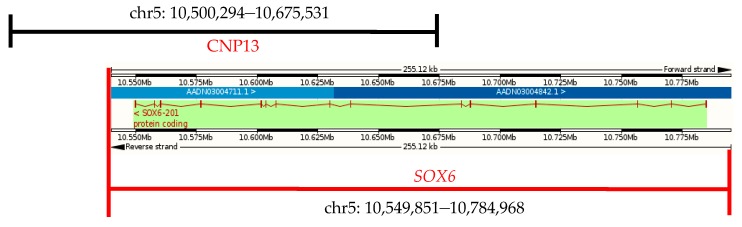
Gene location of chicken *SOX6*.

Chicken *SOX6* is located in chromosome 5 (chr5), with a total length of 179,522 bp, including 15 exons, 14 introns, and the full-length of its coding sequence is 2370 bp. Chicken *SOX6* (Ensemble, Galgal4: CM000097.3) overlaps with the CNP13 (Figure 3). The gene’s CNV occurs in some individuals in both the XH and WRR chicken lines, and *SOX6* may play an important role in the chicken’s growth traits.

### 3.6. The Expression Level of SOX6 is Positively Related to Its Copy Number

**Figure 4:**
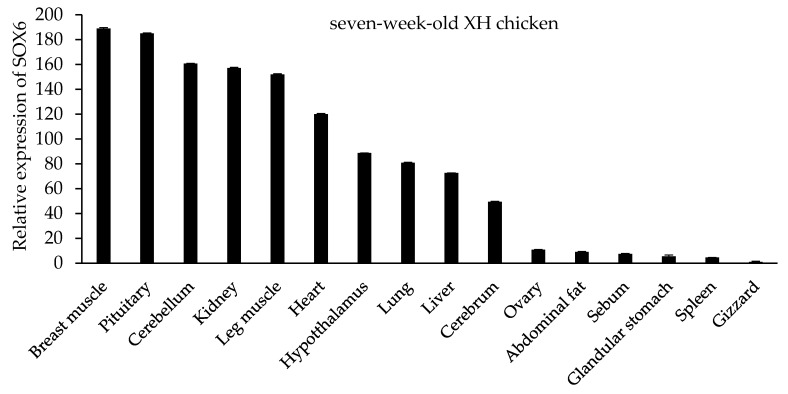
The tissue expression profile of SOX6 in seven-week-old XH chickens.

**Figure 5:**
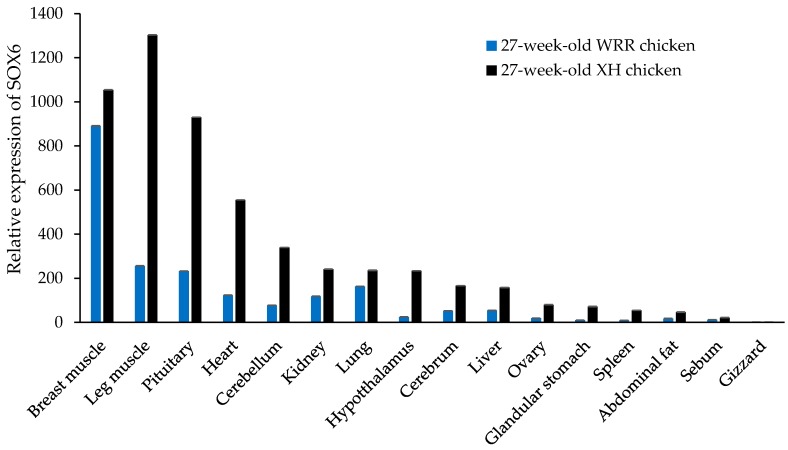
The tissue expression pattern of SOX6 in both 27-week-old XH and WRR chickens.

In seven-week-old XH chickens, SOX6 is expressed in different tissues, such as cerebellum, cerebrum, hypothalamus, pituitary, heart, liver, spleen, lung, kidney, glandular stomach, gizzard, breast muscle, leg muscle, sebum, abdominal fat, and ovary (Figure 4). The highest expression was in the breast muscle and pituitary gland, secondly in the cerebellum, kidney, leg muscle and heart, with lower expression levels in other tissues. Similar to the trend of SOX6 mRNA in different tissues of seven-week-old XH chickens, SOX6 is expressed highly in the breast muscle, leg muscle, pituitary, heart, cerebellum, and kidney of both 27-week-old XH and WRR chickens (Figure 5). 

**Figure 6:**
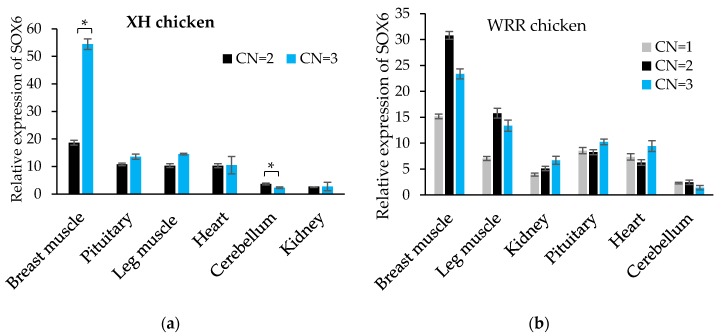
The expression levels of SOX6 in XH and WRR chickens with different copy numbers. (**a**,**b**) The relative expression levels of SOX6 in six tissues of both XH chicken with CN = 2 or 3 and WRR chicken with CN = 1, 2 or 3, respectively. The Significance: * indicates *p* < 0.05.

Additionally, the expression level of SOX6 in the breast muscle of variant XH chickens with CN = 3 was about two times higher than that of the normal individuals (CN = 2), and that in the leg muscle, kidney and heart of the variant individuals was likewise higher than those of normal individuals (Figure 6a). The expression level of SOX6 in the pectoral muscle, leg muscle, kidney and heart of the WRR chickens was increased gradually along with an increase of the copy number (Figure 6b), though differences were not statistically significant. As shown in Table 7, there was a remarkably significant association between SOX6 expression and copy number in breast muscle both in XH and WRR chickens (*p* < 0.01).

### 3.7. Disorder Region of SOX6 Proteins

**Figure 7:**
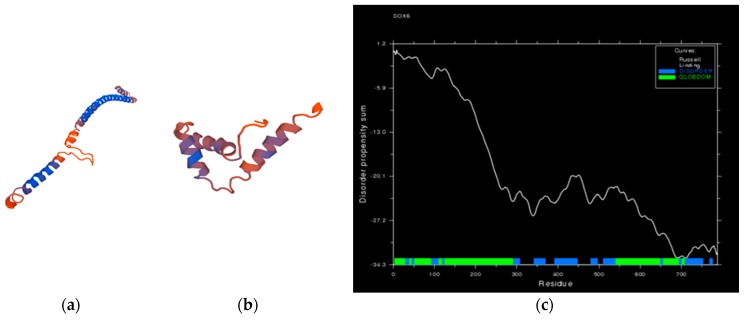
The disorder region of the SOX6 proteins is 265–579 aa. (**a**,**b**) The prediction results of the tertiary structure of SOX6 proteins in 132–264 aa and 580–649 aa were shown on the left and right sides, respectively. (**c**) Prediction of the protein disorder region of the SOX6 proteins. The blue color represents a disordered protein, and green indicates that the protein has a fixed structure.

We used the Swiss model to predict the tertiary structure of SOX6 proteins by homologous construction. The results showed that the 132–264 aa and 580–649 aa regions of the SOX6 proteins had tertiary structures. Its tertiary structure prediction was similar to the secondary structure (Figure 7a,b). However, the proteins’ tertiary structure was unable to be predicted in the 265–579 aa region. From the GlobPlot analysis of the SOX6 amino acid sequence, we found that the 307–508 aa was located in the disordered region and consistent with the prediction results of the Swiss model (Figure 7c). 

### 3.8. Chicken SOX6 Promotes Skeletal Cell Proliferation and Differentiation

**Figure 8:**
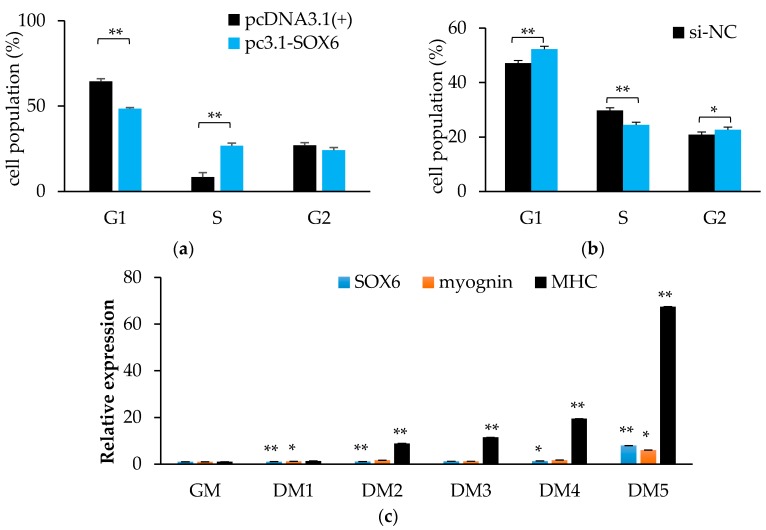
Chicken SOX6 promotes cell proliferation and differentiation. (**a**,**b**) The cell population of cell cycles after overexpressing or interference with SOX6, respectively. (**c**) The expression levels of SOX6, myognin and MHC in QM7 during differentiation medium from one to five days (DM1–5). Significance: *, ** indicate *p* < 0.05 and *p* < 0.01 vs. general medium (GM), respectively.

The homology of SOX6 between quails and chickens is up to 99% (Supplementary Table S1 and Figure S4), therefore, we used QM7 cells to serve as research material. As shown in Figure 8a, cells during the G1 phase were significantly decreased (*p* < 0.01), and while cells in the S phase were remarkably increased (*p* < 0.01) by overexpressing SOX6. We obtained the opposite result from the interference experiment (Figure 8b). In the cells of the general medium (GM), the differentiation medium from one day to five days (DM1–5), the expression level of SOX6 gradually increased (Figure 8c). It demonstrated that chicken *SOX6* plays a positive role in muscle cell differentiation.

**Figure 9:**
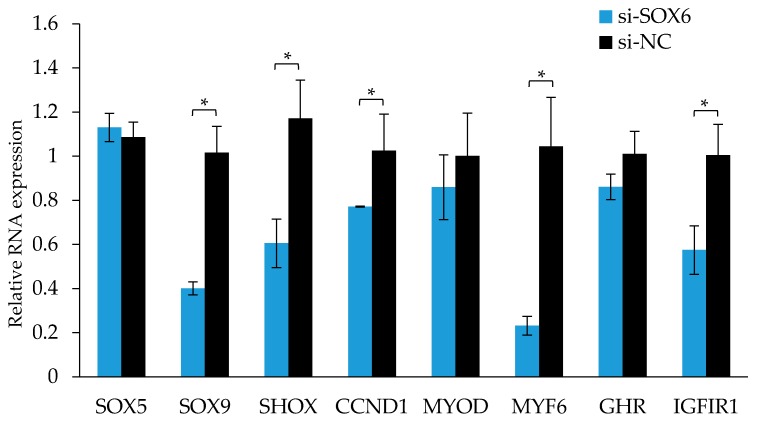
The RNA expression levels of candidate genes after interfering with SOX6 in the QM7 cells. Significance: * indicates *p* < 0.05.

As shown in Figure 9, after the interference of the SOX6, the expression levels of those genes associated with growth and development, such as SHOX, MYF6, and IGFIR were significantly reduced (*p* < 0.05), and the expression levels of those genes associated with cell proliferation, for instance, SOX9 and CCND1 were also significantly reduced (*p* < 0.05).

## 4. Discussion

In order to better reveal the molecular mechanisms of phenotypic genetic variation, an association analysis between a large number of SNPs, genes’ CNVRs and phenotypic traits was carried out [24]. The results show 10 CNPs containing many important chicken-growth-relevant genes, such as *GLCCI1* [25,26], growth hormone receptor gene (CNP19) and *SOX6* (CNP13). CNP13 overlaps with *SOX6*, which cooperated with SOX5 and SOX9 to participate in the development of a variety of organs and tissues [27,28]. We found that the copy number of the CNP13 could increase the expression level of SOX6, suggesting that CNP13 could regulate chicken growth through affecting the function of SOX6.

McCarroll et al. found that 99% of CNV variations are hereditary [2]. The results of CNV genetic law in this study also showed that most of the CNVs followed Mendel’s principles, which were consistent with the genetic characteristics of CNV, though some rare mutations were not consistent with Mendel’s principles. We found that mutations appeared in CNP13 with a gradually decreased frequency of regional deletion in the resource group, and low frequency in other groups. However, the homozygous deletion (0/0) individuals do not appear in the offspring. Since the deletion region probably plays an important function, pure deletion of individuals easily leads to serious abnormalities or disease, likely causing embryonal death, and was eliminated in the process of evolution [29]. An individual with CN = 3 (1/2) was found in this group by CNVplex^®^, which might be generated from the lack of individual variation (0/1) and normal individuals (1/1). Additionally, the repeated events in CNP10 and CNP13 were undertaken by positive selection pressure in evolution, which can be stably transmitted to the offspring. Those with genetic benefits on CNVs may have an important effect on some specific traits, pedigree evolution and formation. Therefore, it is necessary to further study CNVs to find out the candidate CNVs with important functions.

After SOX6 overexpression, a vector was transfected into muscle cells, and the cells in the S phase increased significantly. SOX6 may promote cell growth through promoting cells to transit from the G1 phase to the S phase. To understand the function of SOX6 in cell growth, we detect the expression of key cell-cycle regulatory factors after interfering with SOX6. For instance, *CCND1* can promote cell proliferation, leading to transition tumor progression [30,31]. Insulin-like growth factor 1 (*IGF-1*) can play an important role in chicken growth through promoting DNA synthesis in the cell cycle to promoting cell proliferation [32]. *IGF1R* involves in the regulation of cell cycles, proliferation, differentiation, and metabolism, and its gene polymorphism is related to chicken growth and carcass traits [33,34]. Myogenic regulatory factors (*MRFs*) can regulate cell proliferation and differentiation. Myogenic differentiation antigens (*MYOD*) can promote myoblast differentiation, and have important effects on muscle development [35,36]. Myogenic factor 6 (*MYF6*) enhances the expression of muscle proteins and other related muscle-specific genes through trans-activation [37]. The expression level of *CCND1*, a cell-proliferation-promoting gene, was significantly decreased after the interference of SOX6 (*p* < 0.01). With the knockdown of SOX6, the expression levels of IGF1R and MYF6 was likewise decreased significantly. Chicken *MYF6* is closely related to chicken meat quality and carcass traits [38], and CNP13 is related to leg muscle shear force and dry matter content, the *MYF6* may combine with *SOX6* (CNP13) and participate in chicken muscle growth and meat quality traits. The results showed that the expression levels of SOX9 and SHOX were significantly reduced after the interference of SOX6, suggesting that together these three genes play a similar role in chicken growth. 

Tissue-specific gene expression is closely related to its biological function. Human SOX6 is highly expressed in nerve tissue [39]. However, high expression levels of SOX6 were found in chicken breast muscle, leg muscle, kidney, heart, cerebellum and pituitary. Since muscles and kidneys are closely related to the growth and development of individuals, the high expression of SOX6 in muscles suggests that it might have an effect on muscle growth and development. 

As an important genetic variation, the dose effect of the copy number of a CNV can change gene structure, expression and even affect gene function. Previous results have showed that the gene copy number can cause a change in the expression level of mRNA. Some gene number variations are proportional to gene expression [40], and some are opposite [41]. We found that the mRNA expression of SOX6 in chicken breast muscle with an SOX6 repeat copy number is two times higher than that in normal individuals. In the muscle and kidney tissues of WRR chickens, the mRNA expression of SOX6 is proportional to the copy number of the gene. It is suggested that there might be an enhancer in the region to promote the expression level of SOX6. The number of enhancers is increased followed by an increase in the copy number in CNP13. Thereby, *SOX6* is positively associated with the development of chicken muscle.

It was showed that GlobPlot online software could analyze the ordered or disordered regions of a protein [20]. In this study, from the GlobPlot analysis of the SOX6 amino acid sequence, we found that the 300–580 aa region is a disorder region. Notably, combined with the results of the CNVplex^®^ analysis, a 378–508 peptide segment of SOX6 is located in the disordered region, which indicated that the CNV may be related to the disorder region of the SOX6 proteins. A major determinant of polypeptide segments folding co-operatively into a defined tertiary structure is the long-range hydrophobic interaction between amino acids in the linear sequence. Intrinsically disordered regions (IDRs) are polypeptide segments that do not contain sufficient hydrophobic amino acids to mediate co-operative folding. Instead, they typically contain a higher proportion of polar or charged amino acids [42]. Thus, IDRs lack a unique three-dimensional structure either entirely or in part in their native state. They generally shape a variety of conformations that are in dynamic equilibrium under physiological conditions [43,44]. These properties of IDRs make them well-suited to perform signaling and regulatory functions. Indeed, the functions of proteins with IDRs have revealed that they are enriched in signaling proteins and nucleic acid-binding proteins such as kinases, transcription factors and splicing factors [45,46,47]. In chicken, we found that the CNV of *SOX6* genes resulted in a disorder structure of SOX6 proteins, which may interact with SOX9 and SHOX to repress the G1 phase and up-regulate the S phase in the cell-cycle process, resulting in promotion of cell proliferation and differentiation. However, the limitations of this work were that we cannot clear out what kind of structures *SOX6* genes have with or without CNVs in chicken, and what the different functions are between ordered and disordered SOX6 proteins.

## 5. Conclusions

We found that the expression level of SOX6 was positively increased by the copy number, and the disorder structure of SOX6 proteins could contribute to the development of muscle growth through up-regulating the expression levels of the muscle growth related genes in chickens as in other animal species. 

## Figures and Tables

**Table 1:** The frequency of copy number polymorphisms (CNPs) in Xinghua chicken (XH) **×** white recessive rock chicken (WRR) full sib resource group.

CNP	Chr.	Start	End	CN	Gene	Frequency (%)
CNP1	1	114669300	115092768	0, 1	*EFHC2*, *MAOA*	3.83
CNP2	2	24780291	24949275	1	*GLCCI1*	5.66
CNP3	4	88930573	89076844	3	*CD8A*	3.65
CNP4	10	9320467	10038259	1	*ARPP19*, *KIAA1370*, *MYO5A*	3.65
CNP5	16	220950	299166	0, 3	*B-G*, *LOC396417*, *YFV*	9.49
CNP6	18	3342968	3377149	1	*RAB40B*, *WDR45B*	4.01
CNP7	20	105179	224625	1	*C20orf4*	4.01
CNP8	1	19027876	19083051	3	NOT_FOUND	3.83
CNP9	1	66088686	66170851	1, 0	NOT_FOUND	6.02
CNP10	1	144183468	144308761	3	NOT_FOUND	8.76
CNP11	1	167313565	167378938	1	NOT_FOUND	4.74
CNP12	2	132873944	132955032	0, 1	NOT_FOUND	6.57
CNP13	5	12088601	12242199	1	*SOX6*	6.75
CNP14	8	9713469	9770642	1	NOT_FOUND	4.20
CNP15	2	27005716	27140786	1	NOT_FOUND	3.28
CNP16	2	137594135	137647003	1	NOT_FOUND	3.10
CNP17	8	29199309	29221258	1	NOT_FOUND	3.10
CNP18	13	716593	769986	3	*PCDHA*	3.19
CNP19	Z	12889235	12952470	0	*GHR*	3.19

Chr., Chromosome; CN, copy number.

**Table 2:** Detection of copy number variations (CNVs) in candidate CNPs.

		CNV Frequency (%)							
		Illumine SNP Array				AccuCopy^®^ CNVplex^®^		De-novo ^1^ Sequencing	
CNP	CN ^2^ (Genotype)	XH	WRR	XH × WRR F1	XH × WRR F2	XH	WRR	XH	WRR
CNP2	1 (1/0)	- ^3^	-	-	5.66	-	-	-	-
CNP4	1 (1/0)	7.14	14.29	-	3.40	-	-	-	-
CNP6	1 (1/0)	-	7.14	3.57	4.00	-	-	-	10.00
CNP10	3 (1/2)	-	14.29	17.86	8.20	6.00	-	100.00	100.00
CNP13	1 (1/0)	14.29	7.14	-	6.80	-	1.00	-	-
	3 (1/2)	-	-	-	-	2.00	4.00	4.00	10.00

^1^ quality scores (Q-value) lower than 20 were removed from the raw RNA-Seq data using Perl scripts from Novogene Bioinformatics Technology Co., Ltd. (Beijing, China). At the same time, Q20, Q30, GC-content, and sequence duplication levels of the clean data were calculated. The clean data used in this study. ^2^ CN, copy number. ^3^ “-” indicates none detected.

**Table 3:** Association of CNPs in the XH × WRR full sib F2 resource group and economic traits.

CNP ID	Trait	*p* Value ^1^	Contribution Rate (%)	Mean ± SD ^2^	
CNP14	breast muscle conductivity	0.04675	26.81	8.19 ± 0.67 ^a,3^ (CN = 1)	6.32 ± 0.18 ^b^ (CN = 2)
	breast muscle dry matter content (%)	0.0119	25.93	25.23 ± 0.34 ^B^ (CN = 1)	26.19 ± 0.21 ^A^ (CN = 2)
CNP17	63 d tibia length (mm)	0.0029	52.50	90.67 ± 3.49 A (CN = 1)	76.1 ± 0.69 B (CN = 2)

^1^ *p* value corrected for false discovery rate (FDR), FDR ≤ 0.05 was considered a significant difference. ^2^ Standard Deviation (SD). ^3^ In the same line, different capital letters indicate a remarkable significant difference (*p* < 0.01), different small letters indicate significant difference (*p* < 0.05).

**Table 4:** The functions of the candidate CNPs.

CNP ID	CN	Gene	Up or Down-Regulate Growth Traits
CNP2	1	*GLCCI1*	↑
CNP3	3	*CD8A*	↓
CNP4	1	*ARPP19*, *KIAA1370*, *MYO5A*	↓
CNP6	1	*WDR45B*, *RAB40B*	↑
CNP10	3	*NOT FOUND*	↑
CNP13	1	*SOX6*	↓

CN, copy number. Arrows ↑ and ↓ represent that genes up-regulate or down-regulate chicken growth traits, respectively.

**Table 5:** qRT-PCR results verifying six CNPs.

CNP ID	CNP Region	Type	Validation	Positive Verification Rate (%)
CNP2	chr2: 24780291–24949275	LOSS	Yes	80
CNP3	chr4: 88930573–89076844	GAIN	Yes	100
CNP4	chr10: 9320467–10038259	LOSS	Yes	80
CNP6	chr18: 3342968–3377149	LOSS	Yes	80
CNP10	chr1: 144183468–144308761	GAIN	Yes	100
CNP13	chr5: 12088601–12242199	LOSS	Yes	100

**Table 6:** The result of the AccuCopy^®^ assay.

CNP	Region ^1^	CNV Type		
		XH	WRR	
CNP2	GLCCI1E04	CN = 2	CN = 2	
CNP4	ARPP19E02	CN = 2	CN = 2	
CNP6	RAB40BE03	CN = 2	CN = 2	
	FAM214AE05	CN = 2	CN = 2	
CNP13	SOX6E09	CN = 2	CN = 2	CN = 1 (1%) ^1^

^1^ The frequency in the experimental population.

**Table 7:** The significance of association between *SOX6* RNA expression and copy number (*p*-value).

	Breast Muscle	Pituitary	Leg Muscle	Heart	Cerebellum	Kidney
**XH**	0.0000323436	0.53629558	0.830763012	0.958416629	0.026859996	0.177897854
**WRR**	0.001143708	0.147867296	0.190177707	0.400274735	0.175778229	0.255664008

## Supplementary Materials

The following are available online at www.mdpi.com/2073-4425/9/1/42/s1. File1: Table S1: Homologous alignment of SOX6 coding sequences among 12 different species (%). Table S2: Primers used in analysis of CNP analysis in chicken. TableS S3–S11: Population genetic of CNV in family 1, 5, 7, 8, 10, 13, 15, 16 and 17, respectively. Figure S2: LD analysis between six candidate CNPs and their SNPs in 500 kb on either side. Figure S3: The result of CNVplex^®^ assay using different probes in three CNPs. Figure S4: Phylogenetic tree of SOX6 proteins among different species. File2: The result of CNVplex^®^ assay.
