# Supplementary material for: Copy Number Variation in SOX6 Contributes to Chicken Muscle Development
Source: Genes (Basel). 2018 Jan 17;9(1):42. doi: 10.3390/genes9010042 (PMC5793193; doi:10.3390/genes9010042)
Supplement: Supplementary file 1 [file genes-09-00042-s001.zip › genes-243091-revised supplementary file1.docx]

**Supplementary**

**Table S1.** Homologous alignment of SOX6 coding sequences among 12 different species (%)

|  | **G** | **H** | **P** | **Clf** | **B** | **M** | **R** | **X** | **D** | **Cj** | **E** | **O** |
| --- | --- | --- | --- | --- | --- | --- | --- | --- | --- | --- | --- | --- |
| **G** | 100 | 86 | 86 | 85 | 87 | 84 | 85 | 78 | 80 | 99 | 88 | 89 |
| **H** |  | 100 | 99 | 95 | 96 | 93 | 91 | 79 | 80 | 86 | 97 | 97 |
| **P** |  |  | 100 | 95 | 96 | 93 | 91 | 79 | 80 | 86 | 97 | 98 |
| **Clf** |  |  |  | 100 | 96 | 92 | 90 | 84 | 82 | 84 | 96 | 98 |
| **B** |  |  |  |  | 100 | 93 | 91 | 79 | 79 | 87 | 97 | 99 |
| **M** |  |  |  |  |  | 100 | 97 | 75 | 82 | 85 | 93 | 95 |
| **R** |  |  |  |  |  |  | 100 | 78 | 78 | 85 | 92 | 95 |
| **X** |  |  |  |  |  |  |  | 100 | - | 78 | 78 | 79 |
| **D** |  |  |  |  |  |  |  |  | 100 | 79 | 82 | 79 |
| **Cj** |  |  |  |  |  |  |  |  |  | 100 | 87 | 87 |
| **E** |  |  |  |  |  |  |  |  |  |  | 100 | 98 |
| **O** |  |  |  |  |  |  |  |  |  |  |  | 100 |

G, *Gallus gallus*; H, *Homo sapiens*; P, *Pan troglodytes*; Clf, *Canis lupus familiaris*; X, *Xenopus tropicalis*; B, *Bos taurus*; M, *Mus musculus*; R, *Rattus norvegicus*; D, *Danio rerio*; Cj, *Coturnix japonica*; E, *Equus caballus*; O, *Ovis aries musimon*.

**Table S2**. Primers used in analysis of CNP analysis in chicken

| **Application** | **CNP ID/Gene** | **Primer name** | **Primer sequence (5' to 3')** | **Tm**  **(°C)** |
| --- | --- | --- | --- | --- |
| CNP3 qPCR | CNP3 | CD8A-F | ATGACATCACATCCTCTTCT | 52 |
|  |  | CD8A-R | CCGCTTCCATACTCACAG |  |
| CNP2 qPCR | CNP2 | GLCCI1-F | CGGAATGAGCTATGAGGGCCAA | 59 |
|  |  | GLCCI1-R | TCCTGCTGCATGGTGAACACAA |  |
| CNP4 qPCR | CNP4 | MYO5A-F | CTGCCAACCACCTGACCTT | 59 |
|  |  | MYO5A-R | AACACTTCAAGAGATGCCAACA |  |
| CNP6 qPCR | CNP6 | WDR45B-F | CCCGGATTTTTGATATGGTGTTT | 64 |
|  |  | WDR45B-R | GCGTGAAGCTGGTGCAATTAA |  |
| CNP10 qPCR | CNP10 | CNP10-3F | AAACTCCTCCTCTATCCTGAC | 56.9 |
|  |  | CNP10-3R | CTGAATGCCTTCCTTGTG |  |
| CNP13 qPCR | CNP13 | SOX6E12-F | CCCAATGAAGATGGAAAAC | 55 |
|  |  | SOX6E12-R | CTGGAGCAGCTAGAGGATA |  |
| Internal control in qPCR analiysis | *PCCA* | PCCA2-F | TGAATGCCACGGTGCTCCT | 52-64 |
|  |  | PCCA2-R | ACAGTAAAGAAATGTCGGG |  |
| SOX6 CDS full-length PCR | *SOX6*-1 | X*SOX6*F | ATGTCTTCCAAGCAGGCTACCTCTCC | 62 |
|  |  | X*SOX6*R | TCAGTTGGCACTGACTGCCTCATG |  |
| SOX6 partial mRNA PCR | *SOX6-*2 | SOX6-F | AATTCAGGTTCAGGGTCACAT | 56.9 |
|  |  | SOX6-R | ACTGCACAGGGTAGTTATCAC |  |
| CylinD1 qRT-PCR | *CylinD1* | CyclinD1F | CAGAAGTGCGAAGAGGAAGT | 57.3 |
|  |  | CyclinD1R | CTGATGGAGTTGTCGGTGTA |  |
| MYOD qRT-PCR | *MYOD* | MYOD-F | ATCACCAAATGACCCAAAGC | 57 |
|  |  | MYOD-R | GGGAACAGGGACTCCCTTCA |  |
| MYF6 qRT-PCR | *MYF6* | MYF6-F | CGCCATCAGCTACATCGAGAG | 58 |
|  |  | MYF6-R | CCGCAGGTGCTCAGGAAGT |  |
| GHR qRT-PCR | *GHR* | GHR-F | CCTGATCCCCCTGTGCACCTTA | 60 |
|  |  | GHR-R | GGAACCACTGTTGAGAGCCTGG |  |
| IGF1R qRT-PCR | *IGF1R* | IGF1R-F | TTCAGGAACCAAAGGGCGA | 59 |
|  |  | IGF1R-R | TGTAATCTGGAGGGCGATACC |  |
| SHOX qRT-PCR | *SHOX* | SHOX-2F | TGGCCCCGTACGTCAATATG | 59.4 |
|  |  | SHOX-2R | GCTGGAGTTCTTGCTGTTGC |  |
| SOX5 qRT-PCR | *SOX5* | SOX5-1F | TTGCCATGGGATCAGGGAAC | 59 |
|  |  | SOX5-1R | GATCTGTGAGGCAGCCAGTT |  |
| Internal control in qRT-PCR analiysis | *β-actin* | β-actin -F | CCCCATGCCATCCTCCGTCTG | 54-65 |
|  |  | β-actin -R | CCTCGGGGCACCTGAACCTCTC |  |

**Table S3.** Population genetics of CNV in family 1

| **CNVR** | **Gene** | **Inheritance law of CNV** | | | | | | | | | | |
| --- | --- | --- | --- | --- | --- | --- | --- | --- | --- | --- | --- | --- |
|  |  | **F0♂ × F0♀** | | | | **F1♂ × F1♀** | | | | **F2** | | **Genetic variant from heredity** |
|  |  | **CN** | **Genotype** | **CN** | **Genotype** | **CN** | **Genotype** | **CN** | **Genotype** | **CN** | **Genotype** |  |
| chr1:199251493-199328195 | *CCKBR, ILK, MIR1600* | 2 | 1/1 | 2 | 1/1 | **1** | **0/1** | 2 | 1/1 | 1 | 0/1 | P |
|  |  |  |  |  |  |  |  |  |  | 2 | 1/1 |  |
| chr4:83577454-83674690 | NONE | 2 | 0/2 | 2 | 1/1 | 3 | 1/2 | 3 | 1/2 | 2 | 1/1 | Y |
|  |  |  |  |  |  |  |  |  |  | 3 | 1/2 |  |
| chr6:19539037-19558297 | NONE | 2 | 1/1 | 2 | 1/1 | 2 | 1/1 | **1** | **0/1** | 2 | 1/1 | P |
|  |  |  |  |  |  |  |  |  |  | 1 | 0/1 |  |
| chr10:7102154-7170759 | NONE | 2 | 1/1 | 2 | 1/1 | **1** | **0/1** | 2 | 1/1 | 1 | 0/1 | P |
|  |  |  |  |  |  |  |  |  |  | 2 | 1/1 |  |
| chr13:716593-769986 | *PCDHA* | 2 | 1/1 | 3 | 1/2 | 2 | 1/1 | 3 | 1/2 | 2 | 1/1 | Y |
|  |  |  |  |  |  |  |  |  |  | 3 | 1/2 |  |
| chr17:8569253-8637548 | *PMPCA* | 2 | 1/1 | 2 | 1/1 | 2 | 1/1 | **1** | **0/1** | 2 | 1/1 | P |
|  |  |  |  |  |  |  |  |  |  | 1 | 0/1 |  |

The CNVRs with red were heterozygous deletion (CN = 1, 0/1) and appeared in F1 generation of the family 1. These mutations were not caused by the F0 generation, but can be inherited to the F2 generation. Y: yes; P: partial.

**Table S4.** Population genetics of CNV in family 5

| **CNVR** | **GENE** | **Inheritance law of CNV** | | | | | | | | | | |
| --- | --- | --- | --- | --- | --- | --- | --- | --- | --- | --- | --- | --- |
|  |  | **F0♂ × F0♀** | | | | **F1♂ × F1♀** | | | | **F2** | | **Genetic variant from heredity** |
|  |  | **CN** | **Genotype** | **CN** | **Genotype** | **CN** | **Genotype** | **CN** | **Genotype** | **CN** | **Genotype** |  |
| chr1:66088686-66136083 | NONE | 1 | 0/1 | 2 | 0/2 | 2 | 0/2 | 2 | 0/2 | 0 | 0/0 | Y |
|  |  |  |  |  |  |  |  |  |  | 2 | 0/2 |  |
| chr1:44267367-44349604 | NONE | 2 | 0/2 | 2 | 1/1 | 3 | 1/2 | 3 | 1/2 | 2 | 1/1 | Y |
|  |  |  |  |  |  |  |  |  |  | 3 | 1/2 |  |

The two CNVRs of F1, F2 generation were derived from heredity and in agreement with Mendel's law of segregation.Y: yes.

**Table S5.** Population genetics of CNV in family 7

| **CNVR** | **Gene** | **Inheritance law of CNV** | | | | | | | | | | |
| --- | --- | --- | --- | --- | --- | --- | --- | --- | --- | --- | --- | --- |
|  |  | **F0♂ × F0♀** | | | | **F1♂ × F1♀** | | | | **F2** | | **Genetic variant from heredity** |
|  |  | **CN** | **Genotype** | **CN** | **Genotype** | **CN** | **Genotype** | **CN** | **Genotype** | **CN** | **Genotype** |  |
| chr1:66088686-66136083 | NONE | 1 | 0/1 | 2 | 1/1 | 2 | 1/1 | 2 | 1/1 | 2 | 1/1 | P |
|  |  |  |  |  |  |  |  |  |  | **1** | **0/1** |  |
| chr8:29216125-29247993 | NONE | 2 | 1/1 | 2 | 1/1 | 2 | 1/1 | **1** | **0/1** | 1 | 0/1 | P |
|  |  |  |  |  |  |  |  |  |  | 2 | 1/1 |  |
| chr23:5246857-5303945 | *MIR30C1 MIR30E* | 1 | 0/1 | 2 | 1/1 | 1 | 0/1 | 2 | 1/1 | 2 | 1/1 | Y |
|  |  |  |  |  |  |  |  |  |  | 2 | 1/1 |  |
| chr25:1141355-1163379 | NONE | 2 | 1/1 | 2 | 1/1 | 2 | 1/1 | **3** | **1/2** | 2 | 1/1 | Y |
|  |  |  |  |  |  |  |  |  |  | 3 | 1/3 |  |

The variations in the CNVRs with red in family 7 were not entirely from inheritance, which means that the first and second CNVRs with CN = 1 (0/1), and the fourth CNVR with CN = 3 (1/2) were not caused by heredity. Y: yes; P: partial.

**Table S6.** Population genetics of CNV in family 8

| **CNVR** | **Gene** | **Inheritance law of CNV** | | | | | | | | | | |
| --- | --- | --- | --- | --- | --- | --- | --- | --- | --- | --- | --- | --- |
|  |  | **F0♂ × F0♀** | | | | **F1♂ × F1♀** | | | | **F2** | | **Genetic variant from heredity** |
|  |  | **CN** | **Genotype** | **CN** | **Genotype** | **CN** | **Genotype** | **CN** | **Genotype** | **CN** | **Genotype** |  |
| chr1:199251493-199328195 | *CCKBR ILK MIR1600* | 2 | 1/1 | 1 | 0/1 | 2 | 1/1 | 1 | 0/1 | 2 | 1/1 | Y |
| chr1:144183468-144308761 | NONE | 2 | 1/1 | 2 | 0/2 | 3 | 1/2 | 3 | 1/2 | 2 | 1/1 | Y |
|  |  |  |  |  |  |  |  |  |  | 3 | 1/2 |  |
| chr1:153064381-153139905 | NONE | 2 | 1/1 | 2 | 1/1 | 2 | 1/1 | **3** | **1/2** | 2 | 1/1 | P |
|  |  |  |  |  |  |  |  |  |  | 3 | 1/2 |  |
| chr4:83577454-83674690 | NONE | 2 | 1/1 | 2 | 1/1 | 2 | 1/1 | **3** | **1/2** | 2 | 1/1 | P |
|  |  |  |  |  |  |  |  |  |  | 3 | 1/2 |  |
| chr18:3355021-3377149 | *RAB40B WDR45B* | 2 | 1/1 | 2 | 1/1 | 2 | 1/1 | **1** | **0/1** | 2 | 1/1 | P |
|  |  |  |  |  |  |  |  |  |  | 1 | 0/1 |  |
| chr22:3064289-3110430 | NONE | 2 | 1/1 | 2 | 0/2 | 1 | 0/1 | 1 | 0/1 | 1 | 0/1 | Y |
|  |  |  |  |  |  |  |  |  |  | 2 | 1/1 |  |

The first, second and sixth CNVRs in family 8 were in agreement with Mendel's law of segregation. CNVR variations with red in the F1 generation were not inherited from F0, but can be inherited to the next generation (F2 generation). Y: yes; P: partial.

**Table S7.** Population genetics of CNV in family 10

| **CNVR** | **Gene** | **Inheritance law of CNV** | | | | | | |
| --- | --- | --- | --- | --- | --- | --- | --- | --- |
|  |  | **F1♂ × F1♀** | | | | **F2** | | **Genetic variant from heredity** |
|  |  | **CN** | **Genotype** | **CN** | **Genotype** | **CN** | **Genotype** |  |
| chr16:220950-299166 | *BG YFV* | 2 | 1/1 | 3 | 1/2 | 2 | 1/1 | Y |
|  |  |  |  |  |  | 3 | 1/2 |  |

The variation of CN = 3 (1/2) in the F2 generation 10 was inherited from the F1 generation. Y: yes.

**Table S8.** Population genetics of CNV in family 13

| **CNVR** | **Gene** | **Inheritance law of CNV** | | | | | | | | | | |
| --- | --- | --- | --- | --- | --- | --- | --- | --- | --- | --- | --- | --- |
|  |  | **F0♂ × F0♀** | | | | **F1♂ × F1♀** | | | | **F2** | | **Genetic variant from heredity** |
|  |  | **CN** | **Genotype** | **CN** | **Genotype** | **CN** | **Genotype** | **CN** | **Genotype** | **CN** | **Genotype** |  |
| chr1:114980159-115032581 | NONE | 2 | 1/1 | 1 | 0/1 | 1 | 0/1 | 2 | 1/1 | 0 | 0/0 | P |
|  |  |  |  |  |  |  |  |  |  | 1 | 0/1 |  |
|  |  |  |  |  |  |  |  |  |  | 2 | 1/1 |  |
| chr10:9320467-10038259 | *ARPP19,KIAA1370,MYO5A* | 1 | 0/1 | 1 | 0/1 | 2 | 1/1 | 2 | 1/1 | **1** | **0/1** | P |
|  |  |  |  |  |  |  |  |  |  | 2 | 1/1 |  |
| chr10:7090068-7137602 | NONE | 2 | 1/1 | 1 | 0/1 | 1 | 0/1 | 2 | 1/1 | 1 | 1/1 | Y |
|  |  |  |  |  |  |  |  |  |  | 2 | 1/1 |  |
| chr14:14880297-14905459 | NONE | 2 | 1/1 | 1 | 0/1 | 1 | 0/1 | 2 | 1/1 | 1 | 1/1 | Y |
|  |  |  |  |  |  |  |  |  |  | 2 | 1/1 |  |

The variation in red with CN = 1 (0/1) in family 13 should be CN = 2 (1/1) if it followed the Mendel’s law of segregation. Therefore, this variation was not derived from heredity. Y: yes; P: partial.

**Table S9.** Population genetics of CNV in family 15

| **CNVR** | **Gene** | **Inheritance law of CNV** | | | | | | |
| --- | --- | --- | --- | --- | --- | --- | --- | --- |
|  |  | **F1♂ × F1♀** | | | | **F2** | | **Genetic variant from heredity** |
|  |  | **CN** | **Genotype** | **CN** | **Genotype** | **CN** | **Genotype** |  |
| chr1:144183468-144308761 | NONE | 2 | 1/1 | 3 | 1/2 | 2 | 1/1 | Y |
|  |  |  |  |  |  | 3 | 1/2 |  |
| chr1:167313565-167336723 | NONE | 1 | 0/1 | 2 | 0/1 | 1 | 0/1 | Y |

Two CNVRs in family 15 can be inherited to the next generation. And according to Mendel's law of segregation, the second CNVR may appear in homozygous deletion individual (0/0) in the F2 generation, but there was no CN = 0 individual in this family. Y: yes.

**Table S10.** Population genetics of CNV in family 16

| **CNVR** | **Gene** | **Inheritance law of CNV** | | | | | | |
| --- | --- | --- | --- | --- | --- | --- | --- | --- |
|  |  | **F1♂ × F1♀** | | | | **F2** | | **Genetic variant from heredity** |
|  |  | **CN** | **Genotype** | **CN** | **Genotype** | **CN** | **Genotype** |  |
| chr2:132873944-132955032 | NONE | 0 | 0/0 | 2 | 1/1 | **0** | **0/0** | P |
|  |  |  |  |  |  | 1 | 0/1 |  |
|  |  |  |  |  |  | 2 | 0/2 |  |
| chr2:65829082-65870694 | *RREB1* | 1 | 0/1 | 2 | 1/1 | 1 | 0/1 | Y |
|  |  |  |  |  |  | 2 | 1/1 |  |
| chr2:83121122-83180643 | *GRB10* | 2 | 1/1 | 3 | 1/2 | 2 | 1/2 | Y |

In the F2 generation of family 16, the first CNVR with non-genetic pure and deletion mutation (0/0), the second CNVR with deletion mutation and the third CNV with duplication mutation were inherited from the F1 generation. Y: yes; P: partial.

**Table S11.** Population genetics of CNV in family 17

| **CNVR** | **Gene** | **Inheritance law of CNV** | | | | | | | | | | |
| --- | --- | --- | --- | --- | --- | --- | --- | --- | --- | --- | --- | --- |
|  |  | **F0♂ × F0♀** | | | | **F1♂ × F1♀** | | | | **F2** | | **Genetic variant from heredity** |
|  |  | **CN** | **Genotype** | **CN** | **Genotype** | **CN** | **Genotype** | **CN** | **Genotype** | **CN** | **Genotype** |  |
| chr1:114980159-115032581 | NONE | 2 | 1/1 | 1 | 0/1 | 1 | 0/1 | 2 | 1/1 | 0 | 0/0 | P |
|  |  |  |  |  |  |  |  |  |  | 1 | 0/1 |  |
|  |  |  |  |  |  |  |  |  |  | 2 | 1/1 |  |
| chr10:9320467-10038259 | *ARPP19, KIAA1370, MYO5A* | 1 | 0/1 | 1 | 0/1 | 2 | 1/1 | 2 | 1/1 | 1 | 0/1 | P |
|  |  |  |  |  |  |  |  |  |  | 2 | 1/1 |  |
| chr10:7090068-7137602 | NONE | 2 | 1/1 | 1 | 0/1 | 1 | 0/1 | 2 | 1/1 | 1 | 1/1 | Y |
|  |  |  |  |  |  |  |  |  |  | 2 | 1/1 |  |
| chr14:14880297-14905459 | NONE | 2 | 1/1 | 1 | 0/1 | 1 | 0/1 | 2 | 1/1 | 1 | 1/1 | Y |
|  |  |  |  |  |  |  |  |  |  | 2 | 1/1 |  |

The second CNVR with CN = 1 (0/1) (red) in the F2 generation was not caused by heredity, and the rest were in agreement with Mendel's law of inheritance. Y: yes; P: partial.

| 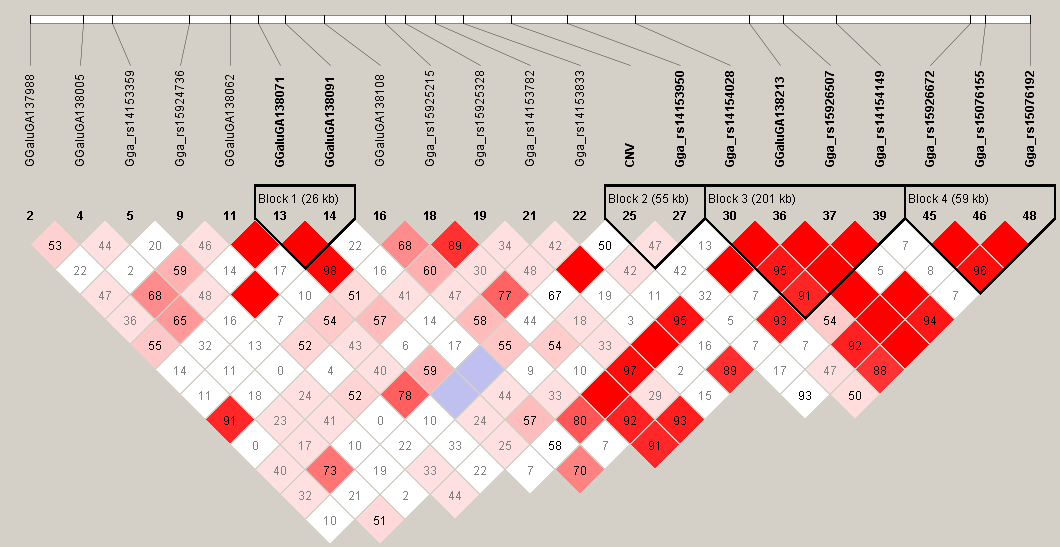   1. LD analysis of CNP2 (chr2:24366111-25404788) and nearby SNPs. |
| --- |
| 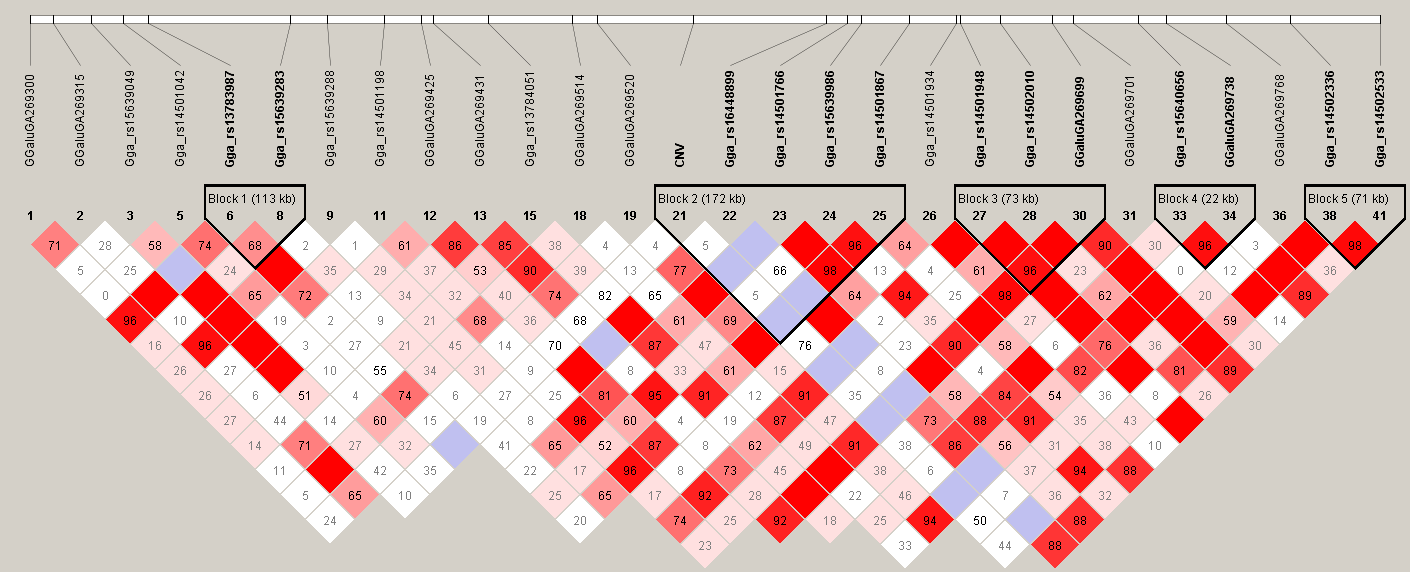   1. LD analysis of CNP3 (chr4:88430573-89576844) and nearby SNPs. |
| 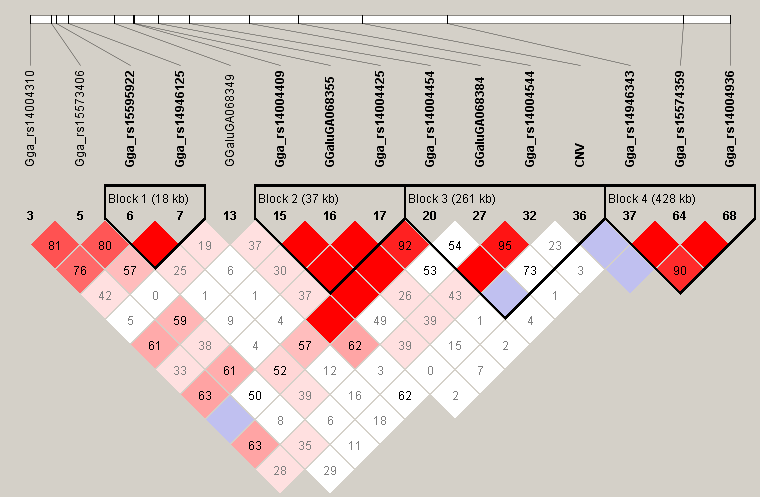   1. LD analysis of CNP4 (chr10: 9349967-10538259) and nearby SNPs. |
| 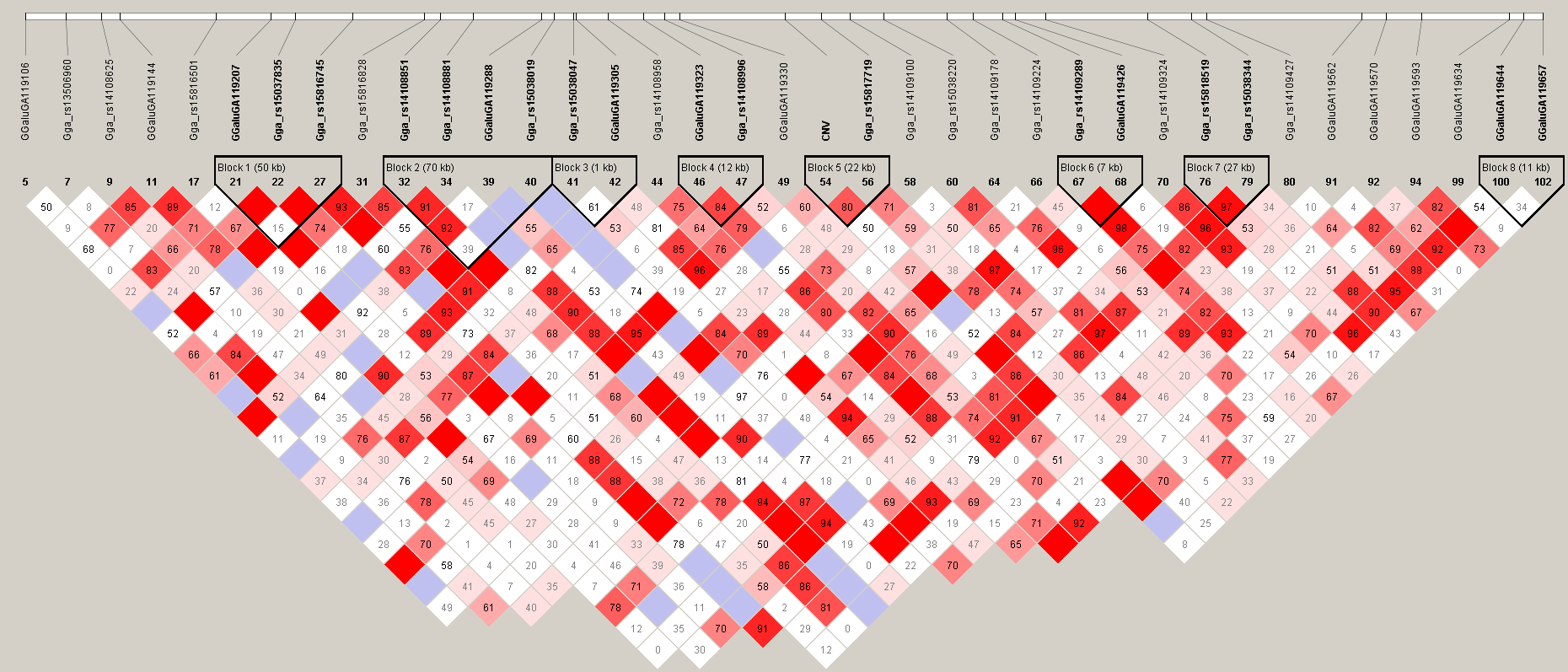   1. LD analysis of CNP6 (chr18:2855021-3877149) and nearby SNPs. |
| 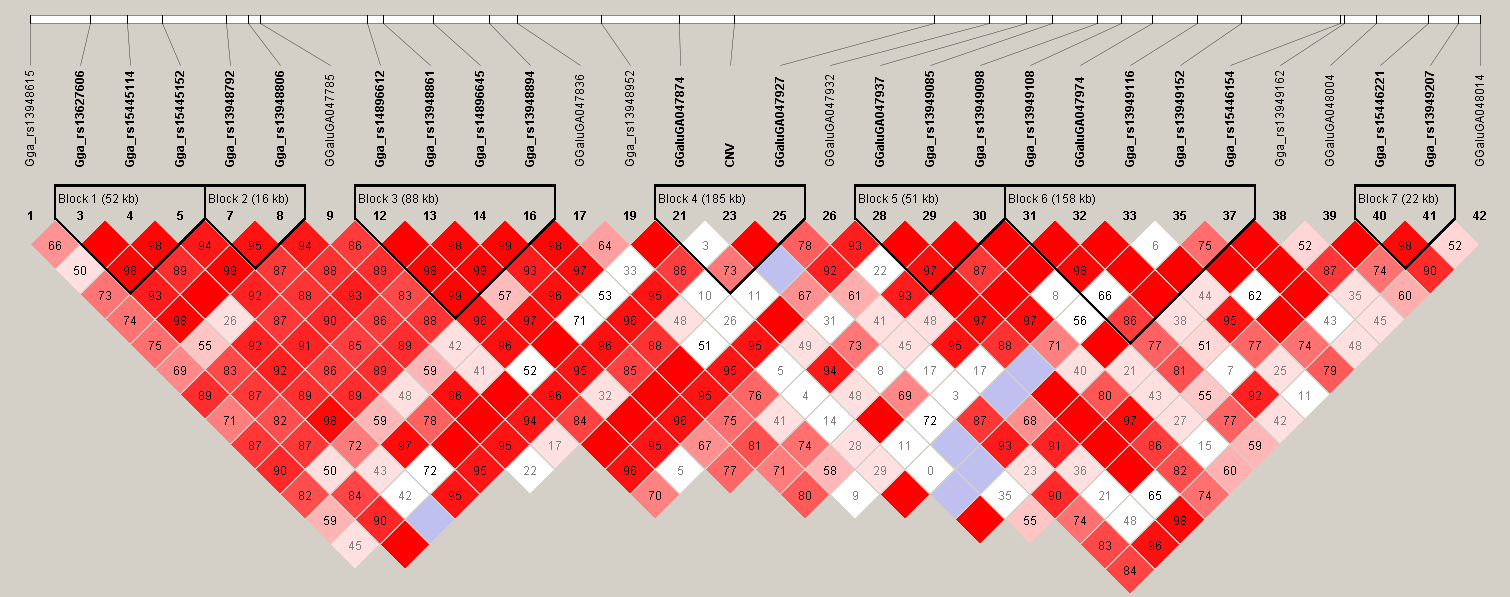   1. LD analysis of CNP10 (chr1:143683468 144758761) and nearby SNPs. |
| 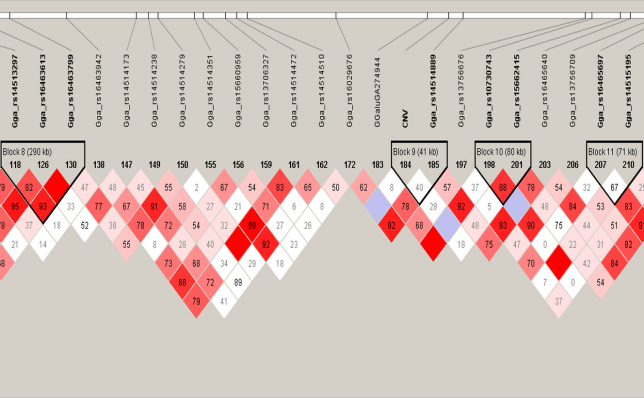   1. LD analysis of CNP13 (chr5:7150295-17242199) and nearby SNPs. |

**Figure S2.** LD analysis of six candidate CNPs and their SNPs in 500 kb on either side.

| 1. CNP4 |
| --- |
| (b) CNP4 |
| (c) CNP6 |
| (d) CNP6 |
| (e) CNP10 |
| (f) CNP10 |
| (g) CNP10 |

**Figure S3.** The result of CNVplex® assay using different probes in three CNPs. Different color lines represent the absolute copy number in different positions of target genes in the experimental individuals. All the absolute copy numbers of *MYO5A* (CNP4) (a,b) and *DR45B* (CNP6) (c,d) ranged from 1.6 to 2.4, which indicated that all the individuals were normal (CN=2). (e–g) The absolute copy number of WRR chicken ranged from 1.6 to 2.4, which demonstrated that all of them were normal individuals (CN=2); however, in XH chicken, there were six individuals’ absolute copy numbers > 2.7 in the 23-26 kb region of CNP10, indicating that all of them were copy number repeat (CN=3) individuals.

| 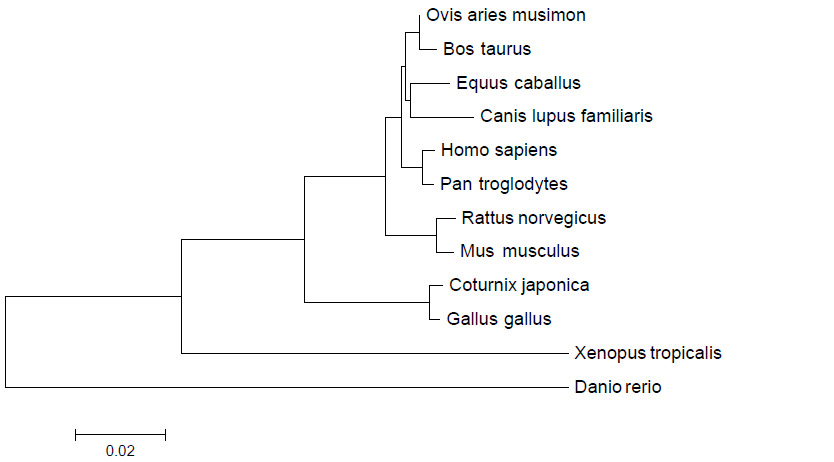 |
| --- |

**Figure S4.** Phylogenetic tree of SOX6 proteins among different species.
